# Red Seaweed (Rhodophyta) Phycocolloids: A Road from the Species to the Industry Application

**DOI:** 10.3390/md22100432

**Published:** 2024-09-25

**Authors:** Madalena Mendes, João Cotas, Diana Pacheco, Kay Ihle, Alina Hillinger, Miguel Cascais, João Carlos Marques, Leonel Pereira, Ana M. M. Gonçalves

**Affiliations:** 1 Marine Resources, Conservation and Technology, Marine Algae Lab, CFE—Centre for Functional Ecology—Science for People & Planet, Department of Life Sciences, University of Coimbra, 3000-456 Coimbra, Portugaljcotas@uc.pt (J.C.); diana.pacheco@uc.pt (D.P.); leonel.pereira@uc.pt (L.P.); 2 IMBRSea, Ghent University, Krijgslaan 281/S8, 9000 Ghent, Belgium; 3 Higher Institute for Tourism and Hotel Management of Estoril, Av. Condes de Barcelona, No. 808, 2769-510 Estoril, Portugal; 4 MARE—Marine and Environmental Sciences Centre/ARNET-Aquatic Research Network, Department of Life Sciences, University of Coimbra, Calçada Martim de Freitas, 3000-456 Coimbra, Portugal; jcmimar@ci.uc.pt; 5 Department of Biology and CESAM, University of Aveiro, 3810-193 Aveiro, Portugal

**Keywords:** polysaccharide, red algae, yield and quality, extraction technology

## Abstract

Seaweed polysaccharides are versatile both in their functions in seaweed physiology and in their practical applications in society. However, their content and quality vary greatly. This review discusses the main factors that influence the yield and quality of polysaccharides, specifically carrageenans and agars (sulfated galactans) found in red algae species (Rhodophyta). In addition, its historical, current, and emerging applications are also discussed. Carrageenan has been influenced mainly by photosynthetically active radiation (PAR) and nitrogen, while its relationship with temperature has not yet been replicated by recent studies. Agar’s seasonal trend has also been found to be more ambiguous than stated before, with light, temperature, nutrients, and pH being influencing factors. In this review, it is also shown that, depending on the compound type, seaweed polysaccharides are influenced by very different key factors, which can be crucial in seaweed aquaculture to promote a high yield and quality of polysaccharides. Additionally, factors like the extraction method and storage of polysaccharides also influence the yield and quality of these compounds. This review also highlights the drawbacks and inadequacy inherent from the conventional (or current) extraction technology approaches.

## 1. Introduction

Under the sea surface, providing a significant part of the world’s oxygen and forming habitats teeming with life, seaweeds grow abundantly. Seaweeds are photoautotrophic multicellular organisms (mainly marine, with some species that live in freshwater) belonging to the domain Eukarya and the kingdoms Plantae (green and red algae) and Chromista (brown algae). Seaweeds are divided into three phyla according to their pigment composition and chemical content [1,2]: green (Chlorophyta), brown (Ochrophyta, Phaeophyceae), and red (Rhodophyta). They are widely distributed geographically, from tropical to polar regions, with ecoregions ranging from intertidal to submerged zones that are still exposed to sunlight [3]. Seaweeds’ chemical content diversity results from their distribution, which translates into a diversity of environments. In response to harsh environmental stresses, seaweeds synthesize unique polysaccharides, long-chain polymers made up of simple sugars that are chained together with glycosidic bonds. These polysaccharides have several functions, such as protection against waves and desiccation, the maintenance of ionic equilibrium, structure for the cell walls, and food reserves [4,5].

Polysaccharide content and quality in seaweeds vary greatly with seasons and are influenced by many different biotic and abiotic factors [6,7,8]. The body of knowledge about factors influencing the polysaccharides in seaweeds varies between the type of polysaccharide; but even for the well-studied ones, conclusions are far from consistent between studies and species. Some reviews exist for the responses of carrageenan [6,9] and agar [8,10], but the great variation in the results of recent studies demonstrates the relevance of a new review for the responses of these polysaccharides in seaweeds.

Abdul Khalil et al. [11] noted that one of the drawbacks of the current phycocolloid production (derived from red seaweed) is the difficulty of controlling and optimizing the yield of agar and carrageenan. Moreover, viable large-scale production benefits from standardized products, as argued for high-value bioactive products derived from seaweeds [12]. Cultivation methods are potentially adjustable to improve polysaccharide content [12]. The control over environmental factors in order to do this can easily be provided in onshore aquaculture [13]. Offshore and nearshore cultivation can be adjusted by selecting locations with both suitable environmental conditions [12] and cultivation techniques [14]. Additionally, knowledge about the seasonality of polysaccharides might also inform the optimal moment of harvest. In addition to practical purposes, understanding the influence of various factors on polysaccharides in seaweeds could be scientifically relevant in our precise understanding of the responses of seaweeds to global warming [15]. All in all, knowledge is needed about the influence of nutrients, carbon metabolism [16], and other influential factors [6,8].

Therefore, the aim of this review is to discuss the main factors that influence the quantity and quality of agar and carrageenan in red algae species (Rhodophyta). Additionally, the chemical structure of these polysaccharides will be emphasized, alongside their historical, current, and emerging applications. Factors like the extraction method and storage of polysaccharides also influence the yield and quality of these compounds, so some highlights of the drawbacks and inadequacy inherent from the conventional (or current) extraction technology will be stated. Finally, abiotic and biotic factors that affect live seaweeds and are relevant for its cultivation are discussed comprehensively in this review.

## 2. Red Seaweed Polysaccharides: Agar and Carrageenan

Humans have used seaweeds for hundreds of years as a food source [17]. Although up to 40% of seaweed production is directly for food, a significant proportion of global seaweed is produced with the aim of deriving polysaccharides [18]. Four widely used seaweed polysaccharides are carrageenan, agar, alginate, and fucoidan [19]. The first three are the main phycocolloids, seaweed-derived (‘phyco’) substances, that can form gels or viscous dispersions with water (‘colloid’) [11,20]. Because of this property, they are commonly used in both the food and pharmaceutical industries, and they represent by far the largest market share of all seaweed polysaccharides [21,22]. The seaweed polysaccharide market is expected to keep on increasing significantly for all four of the above polysaccharides [21]. A telling illustration of this is the boom of Indonesia’s seaweed industry in recent years, which almost exclusively consists of carrageenan-producing seaweeds (carrageenophytes) [13,21].

### 2.1. Chemical Composition

Seaweed polysaccharides are complex sugars present in many types of seaweed. These types of polysaccharides are noteworthy for their unique chemical composition and functional capabilities, which have uses in food, medicines, and biotechnology. Seaweed contains several forms of polysaccharides, including agar, carrageenan, alginate, fucoidan, and laminarin. Here is a breakdown of the chemical makeup of these essential polysaccharides.

#### 2.1.1. Agar

**Figure 1:**
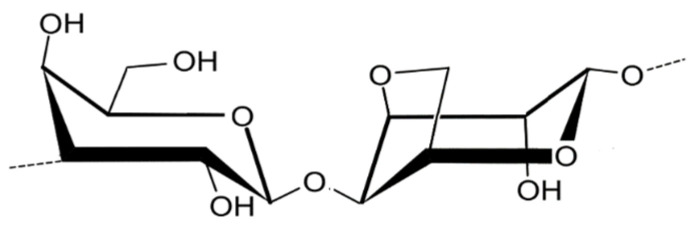
Chemical structure of agar.

Agar (Figure 1) is a polysaccharide mainly found in red algae families such as Gracilariaceae and Gelidiaceae. The main components of agar are agarose and a charged fraction called agaropectin [23]. These two polysaccharides have the same monomers, but different structures. The first one is a linear polymer consisting of alternating β-D-galactose and 3,6-anhydro-L-galactose units linked by glycosidic bonds, and it is the fraction that mostly determines the gelling properties of agar. The second agar component, agaropectin, is a heterogeneous agarose consisting of the same repeating units in which some 3,6-anhydro-L-galactose rings are replaced by L-galactose-6-sulphate or by methoxy or pyruvate groups, consequently reducing the polymer gelling properties. Due to its components, agar is a hydrophilic colloid that can form reversible gels after being cooled from a hot aqueous solution [24,25].

#### 2.1.2. Carrageenan

**Figure 2:**
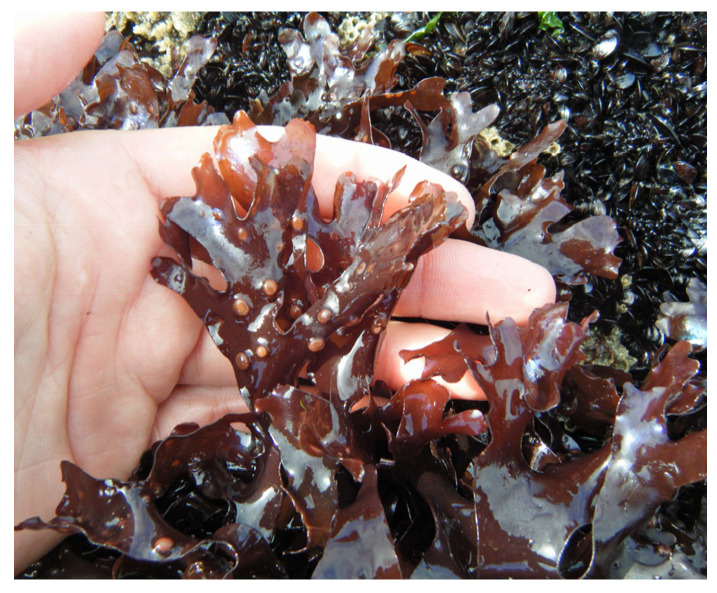
*Chondrus crispus*.

**Figure 3:**
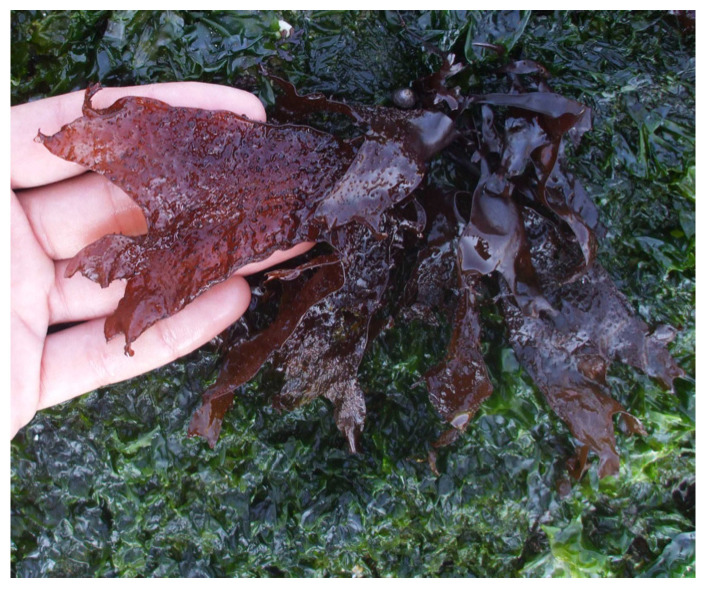
*Mastocarpus stellatus*.

Carrageenan, a polysaccharide only present in red algae, consists of D-galactose and 3,6-anhydo-D-galactose and can reach up to 30–50% of their dry weight [26] in species like *Chondrus crispus* (Figure 2) [27] and *Mastocarpus stellatus* (Figure 3) [28]. The chemical structure of carrageenan is very heterogeneous.

**Figure 4:**
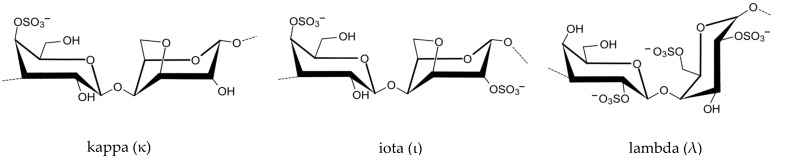
Chemical structure of kappa, iota, and lambda carrageenan.

According to the number and position of sulfate substitutions, as well as the location of 3,6-anhydro bridge in α-1,4-linked galactose residues, the carrageenan polysaccharides can be divided into kappa (κ-), iota (ι-) and lambda (λ-) type carrageenans (Figure 4) [29], although there have been reports of other types like xi, mu, and theta [30]. Carrageenan provides anion-hosting positions for enhanced gel structure because of its double helix arrangement. The different fractions differ in the ions they accept, with κ-carrageenan usually accepting monovalent ions (like K^+^) and ι-carrageenan commonly accepting divalent ions (such as Ca^2+^).

### 2.2. Historical Background

**Figure 5:**
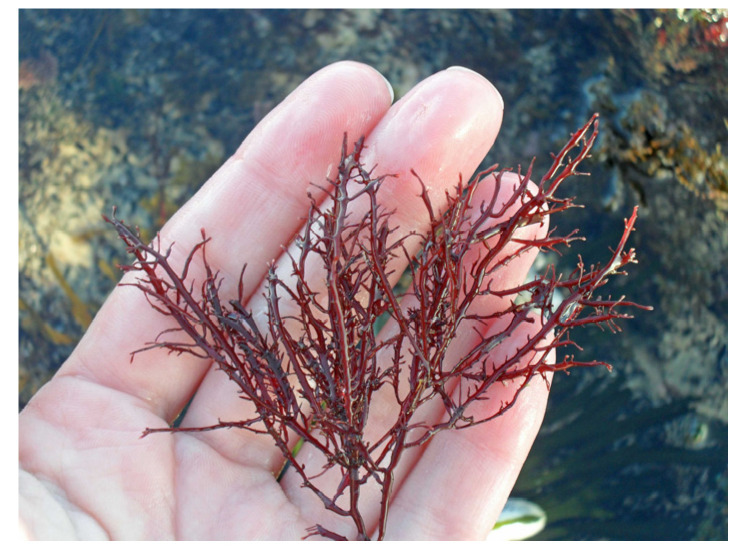
*Gelidium*.

**Figure 6:**
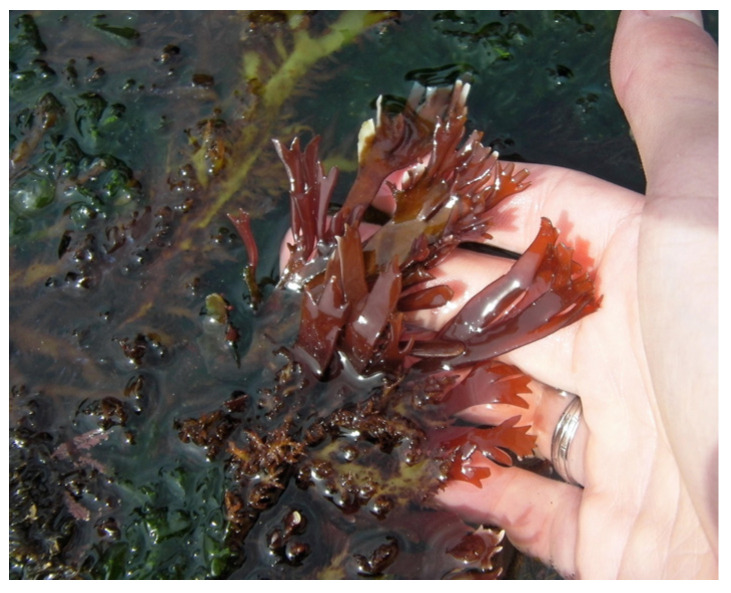
*Gracilaria*.

Seaweeds have been used by humans in the food industry and in medicine for over six centuries, but only relatively recently have natural compounds from them been isolated, identified, and studied [31]. Agar was the first phycocolloid that was extracted, with records of extraction from Mino Tarozaemon in Japan that date back to the late 1650s or early 1660s. In those records, the compound was designated as ‘kanten’ [32]. Nowadays, agar is mostly extracted from seaweeds belonging to the two genera, *Gelidium* (Figure 5) and *Gracilaria* (Figure 6) [33], and due to its thermal reversibility, is primarily used for its thickening properties and serves as a plant-based alternative to animal-origin gelatine [34,35].

Another polysaccharide that is a key ingredient for the food industry is ‘carrageen,’ as it was first called, which was discovered by the British pharmacist Stanford in 1862, who extracted it from the Irish Moss (*Chondrus crispus*). The name was later changed to carrageenan so as to comply with the ‘-an’ suffix for the names of polysaccharides [36]. Carrageenan has been used in Ireland since 400 AD as a gelatine and as a home remedy to cure coughs and colds, and it is known to have been used in China since around 600 BC [37,38], being introduced to the industry in the early 1930s. The modern carrageenan industry dates from the 1940s, after carrageenan being chosen as a stabilizer for the suspension of cocoa in milk chocolate [29].

## 3. Seaweed Polysaccharides’ Current Industrial Applications

Seaweed polysaccharides offer a wide variety of uses due to their distinct functional characteristics. These numerous uses illustrate the value of seaweed polysaccharides in a variety of sectors, owing to their functional qualities such as gelling, thickening, stabilizing, and bioactivity. Although, there are only four major industries (food, pharmaceutical, cosmetic, and agricultural) using a large amount of the agar and carrageenan extracted by the phycocolloid extraction industry.

### 3.1. Food Industry

Agar was the first hydrocolloid, with a European registration number of E406, approved as GRAS (Generally Recognized as Safe) by the Food and Drug Administration (FDA), which employed it as additive in the food industry [39]. About 80% of the agar produced globally is used for food applications [40]. In Asian countries, agar is a popular component of jellies, wherein seaweeds undergo boiling, with some flavour added and cooling to formed jellies. It has been reported that agar applications are based on their functional qualities such as gel strength. Low-quality agar is used basically in food products, while some may extend to industry applications for paper sizing, coating, adhesives, textile printing/dyeing, casting, and impression [11]. Despite being listed as a permitted food additive, agar (E406) is only used in a restricted number of food items. It is believed that around 90% of marketed agar is intended for the food sector [41].

Carrageenan is often applied in dairy products and within the baking industry due to its excellent functional properties. In addition to its properties of binding milk molecules, it also retains water, through which it stabilizes processed meat products. Furthermore, the common use as a jellifying agent is also applied to carrageenan [34,42]. However, it should be noted that its gel-forming abilities depend on the type of carrageenan used, with iota and kappa carrageenan forming a gel in the presence of potassium or calcium [37]. Carrageenans are employed in a variety of food and beverage recipes to provide thickness, stability, and gelling. These phycocolloids are also used for immobilizing biocatalysts, in toothpaste as a stabilizer, in air freshener gels, in pet food, and in meat products [41]. The new applications of carrageenan in the food industry include its use as a protective coating on fresh-cut packaged food, wherein carrageenan acts as a gas barrier, changing the cut surfaces of the fruit and reducing respiration, which consequently slows down the discolorations and maintains texture throughout the shelf life [11,33].

### 3.2. Pharmaceutical and Medicine Industry

In addition to their use in the food industry, algal polysaccharides are commonly used in the pharmaceutical industry and the medical sector, where they are also often used because of their jellifying, stabilizing, and thickening properties, as well as the aforementioned bioactivities [43]. Phycocolloids can be used as ‘functional foods,’ which have health benefits and can prevent chronic diseases [44]. The bio-nanostructures of algal polysaccharides have become a relevant addition to the food industry [45]. Their application is mainly health focused and includes supplements and prebiotics [45,46]. Algal polysaccharides provide antibacterial [47], antimicrobial [48], and antiviral properties [49]. Their medical applications are vast, with algal polysaccharides displaying cancer- and tumor-preventive, anti-inflammatory, and antioxidant properties [50,51]. They are commonly used in drug delivery and disinfection [30,35], while less commonly, they are used as dissolving bandages and in tissue engineering [35,48]. The various polysaccharides also have some specific uses.

Agar is known to decrease blood glucose, which is important for people with hyperglycaemia, and prevents the aggregation of red blood cells. It also has the ability of absorbing ultraviolet radiation [44]. It is also used to produce pharmaceutical-grade growth media for laboratory purposes. Agar is normally used for culture medium because it is not easy to metabolize, is non-digestible, and has good gel firmness, elasticity, clarity, and stability [11]. This kind of agar, usually referred to as medium-quality agar, can be used to obtain monoclonal antibodies, interferons, steroids, and alkaloids, plus act as a bulking agent, laxative, suppository, capsule, tablet, and anticoagulant in medicinal/pharmaceutical fields [11,52]. Meanwhile, the most highly purified agar, usually obtained from a fraction of agar called agarose, is used for separation in molecular biology (electrophoresis, immune diffusion, and gel chromatography) [11,52].

Carrageenan possesses several pharmaceutical properties, such as anticoagulant, antithrombotic, antiviral, antitumor, and cholesterol-lowering effects, as well as immunomodulatory and antioxidant activities [11,52]. The application of carrageenan in the pharmaceutical field was mostly based on these pharmaceutical aspects. Carrageenan is used in the production of tetracyclines, a very important group of antibiotics: With its ability to form a gel, it is used as an immobilizer of *Streptomyces aureofaciens*, the bacteria used for those antibiotics [37]. However, carrageenan can also be used in the production of D-aspartic acid for semi-synthetic antibiotics [37,53]. Moreover, carrageenan has been proven to inhibit the attachment of viruses such as the human papillomavirus, dengue virus, influenza A, and herpes virus [11,52].

### 3.3. Cosmetic Industry

Consumers are increasingly substituting seaweed-based cosmetics for synthetic equivalents. These seaweed-based products often comprise pure components or extracts of several substances. Furthermore, seaweed chemicals have crucial properties for cosmetic use, such as minimal cytotoxicity and allergen concentration. However, seaweeds’ biochemical profiles can vary, and extraction procedures might result in the loss of certain biomolecules. Macroalgal hydrocolloids, also known as phycocolloids, are the most important polysaccharides for industrial commercialization. Phycocolloids are structural polysaccharides found in seaweed that often produce colloidal solutions, which are transitional phases between solutions and suspensions. As a result, polysaccharides may be employed in a variety of sectors, including cosmetics, as thickening, gelling, and stabilizing agents for suspensions and emulsions [54].

Pharmacy and cosmetics consume 20% of total carrageenan output. Carrageenan is found in the formulations of many everyday cosmetic goods, including toothpastes, hair wash products, lotions, medications, sun blocks, shaving creams, deodorant sticks, sprays, and foams [32,55,56,57]. Their ability to form hydrogels enables them to be employed in a variety of applications, including antiviral, antibacterial, and even pathophysiological processes such as hyperlipidemia management [54]. Agar can be used as an emulsifier and stabilizer in creams, as well as to manage the moisture content in cosmetic products such as hand lotions and liquid soap, deodorants, foundation, exfoliant, cleanser, shaving cream, face moisturizer/lotion, and acne and anti-aging treatments [32,58]. Agar can endure high temperatures (up to 250 °C) and preserve its properties even near boiling-point temperatures, making it excellent for its application in jellied confections because the components may be treated at high temperatures and subsequently cooled down [54].

### 3.4. Agriculture Industry

Seaweed polysaccharides are utilized as a functional component in traditional fertilizers to help retain water and nutrients in soils. Their principal use in agriculture is as a soil conditioner. They are natural materials that can absorb massive volumes of water (super-absorbents) at up to hundreds of times their own weight. In agriculture, these polysaccharides are frequently referred to as moisture-holding hydrogels, which improve soil water retention, a critical soil characteristic. Agricultural researchers created super-absorbents to improve soil’s abiotic characteristics. They promote water retention, water use efficiency, soil permeability, infiltration rates, plant performance, and soil aeration. Furthermore, they can reduce irrigation frequency and compaction shift, limit erosion and water drainage, and reduce fertilizer solubility rates [59].

Carrageenans and their oligomers, derived from diverse red seaweeds, are a rich source of bioactive compounds that activate plant defense systems and provide resistance to abiotic and biotic stressors. This can be accomplished by regulating a variety of physiological and biochemical processes. Carrageenans also regulate a variety of plant metabolic processes, including cell division, purine and pyrimidine synthesis, nitrogen and sulfur absorption, and photosynthesis. On the other hand, while numerous studies have been conducted on the bioactivities of agarophytes, there has been little research on the bioactivities of agar, particularly its influence on plants [59].

Thus, the red seaweed polysaccharides have widespread industrial applications nowadays (Table 1). And before introducing the polysaccharides in new industries and usages, there is the need to produce more seaweed with high polysaccharide yield, but with quality secured. Thus, it is important to understand the drivers for seaweed polysaccharide production. Other uses are being developed and applied, for example, in paper sizing, coating, adhesives, textile printing/dyeing, casting, and impression.

Polysaccharide production, notably from red seaweeds, is influenced by a variety of variables, including economic, environmental, technical, and consumer demands [60]. The growing consumer preference for natural and clean-label products is boosting demand for seaweed-derived polysaccharides such as agar and carrageenan. Polysaccharides are useful for controlled drug delivery systems, wound dressings, and tissue engineering due to their biocompatibility, non-toxicity, and gel-forming properties. The designation of polysaccharides as GRAS (Generally Recognized as Safe) by food and drug regulatory agencies such as the FDA expands their usage in a variety of sectors, promoting more manufacturing. Countries with extensive seaweed resources are increasing polysaccharide production to fulfill global demand, particularly in areas with a high consumption of natural and health-promoting components. Seaweed farming is considered more sustainable than land-based agriculture since it uses fewer resources. This sustainability issue is driving increased investment in seaweed polysaccharide synthesis [13].

## 4. Drivers of Polysaccharide Production

Seaweed polysaccharides have been highlighted as a sustainable resource for the future, resulting in an increased demand for their utilization and, subsequently, production. The global need for significant amounts of seaweed will increase in the next years; nevertheless, there is still an ongoing cultivation system optimization required to meet this expanding demand, as well as to ensure sustainable seaweed production and processing. There is a need to grow and collect more seaweed in order to meet the increased demand for seaweeds and seaweed-based goods. The scarcity of farmed seaweed poses a significant threat to wild seaweed populations owing to commercial overexploitation, raising serious marine environmental problems [13]. Thus, for the seaweed polysaccharide industry, it is very important to maintain the quality, but obtain higher yields of the polysaccharides. For this, there is a need to study the seaweed metabolism and reaction to abiotic and biotic factors, and to increase the polysaccharide yield without losing quality value or increasing the economic costs beyond effectiveness.

Many environmental and biological variables affect the formation of seaweed polysaccharides. Knowing and improving these dynamics might help increase seaweed polysaccharide output to satisfy rising global demand while maintaining sustainability and environmental protection. A variety of environmental and biological factors determine the amount, quality, and content of the seaweed polysaccharides produced.

Due to the direct effect of environmental extrinsic variables, seaweed polysaccharides can be synthesized in large quantities and/or at excellent quality. Extrinsic influences on seaweed phenolic compound quality and quantity include seaweed geolocation, ecological characterization, season, biotic factors (herbivory or direct competition with other benthic organisms), and abiotic factors (salinity, pH, light incidence, temperature, and water nutrient composition). However, inherent drivers in seaweed DNA and codifications have a significant influence, perhaps limiting natural polysaccharide synthesis. There are significant variances in the polysaccharides generated by red seaweeds, including their natural amount and innate bioactivities. This explains why various outcomes might be obtained using the same abiotic and biotic factors. Thus, it is necessary to conduct preliminary investigations on seaweed farms for polysaccharide extraction in order for the farms to be economically sustainable and viable [61,62,63]. However, there is scarce literature on this important theme.

### 4.1. Abiotic Factors

Light has a critical role in photosynthesis (carbon sequestration for energetic compounds), influencing seaweed growth and metabolism. The intensity and quality of light can influence the formation of polysaccharides such as agar, carrageenan, and alginate. Seaweed grows at different depths that receive variable levels of light, which influences the amount and kind of polysaccharides produced. Red seaweed flourish in lower light circumstances than green and brown seaweed. Temperature affects seaweed’s metabolic rate and polysaccharide production. The optimal temperature varies by species, although higher temperatures usually enhance growth rates until stress reduces polysaccharide output. Seasonal temperature variations can cause oscillations in polysaccharide content, with certain species generating more during cooler seasons [64,65,66].

These nutrients are needed for seaweed development. Nutrient-rich waters often encourage faster development rates and can alter polysaccharide compositions. However, extra nutrition might cause alterations in the polysaccharide structure. Iron, magnesium, and potassium are also required for the enzymatic activities involved in polysaccharide production. Variations in salinity can cause osmotic stress in seaweed, altering metabolism and polysaccharide formation. High salt levels often enhance the formation of some polysaccharides, such as alginate, as a defensive measure. The kind and number of polysaccharides produced by different seaweed species vary depending on their salinity tolerance. The mechanical motion of water, or water flow, influences nutrient availability and gas exchange, both of which are necessary for seaweed growth and polysaccharide production. Moderate water flow is normally favourable, but excessive circumstances might result in physical damage and reduced polysaccharide output. High turbidity can decrease light penetration, affecting photosynthesis and, hence, polysaccharide formation [64,65,66].

### 4.2. Biotic Factors

Different seaweed species, and even different strains of the same species, have distinct genetic makeup that influences the kind and quantity of polysaccharides produced. Thus, the same seaweed in one location can have distinct input from identical abiotic factors. The stage of the seaweed’s life cycle (i.e., juvenile vs. mature) can alter polysaccharide content, with some stages producing more. Polysaccharide production requires specialized enzymes, which are influenced by both genetic and environmental influences. The activity of these enzymes varies, influencing the amount and structure of the polysaccharides generated [64,65,66,67].

Herbivory and pathogen assaults can also cause polysaccharide synthesis as a defensive strategy. The content of these polysaccharides can vary to improve their protective properties. Microbial populations on the seaweed’s surface can impact polysaccharide synthesis [64,65,66,67].

### 4.3. Red Seaweed Polysaccharide Studies in Polysaccharide Production

#### 4.3.1. Carrageenan

Carrageenan is a sulphated galactan found in the cell walls of various red algae and is thought to give structure to seaweeds, contribute to ionic equilibrium, and protect against desiccation [6]. Véliz et al. [6] conducted a meta-analysis of factors influencing the carrageenan content of Gigartinales seaweeds, based on the carrageenan yields of 63 field research papers and a database of the values of several environmental variables. However, since then, several new studies have been published [15,31,68,69]. Moreover, the meta-analysis did not allow for the consideration of potentially influential factors like wave-action, desiccation and pH, or data from experimental studies. To fill in the knowledge gaps, these subjects will be reviewed here. Genus and ecoregion were the most important predicting factors for carrageenan content [6], which shows the influence of phylogeny and the particular circumstances caused by their direct environment. Furthermore, it underlines that differences in chemical content can be caused by the local adaptation or acclimatization of seaweeds [12]. In addition to genus and ecoregion, the factors were ranked starting with photosynthetically active radiation (PAR), followed by nitrate, temperature, phosphate, depth, salinity, extraction method, family, season, and life cycle phase [6]. Notably, season was ranked among the least important predictors, as was life cycle phase [6], although the type of carrageenan (kappa, iota, or lambda) in Gigartinaceae and Phyllophoraceae was, in fact, found to be dependent on life stages [70].

##### Effect of Photosynthetically Active Radiation (PAR)

**Figure 7:**
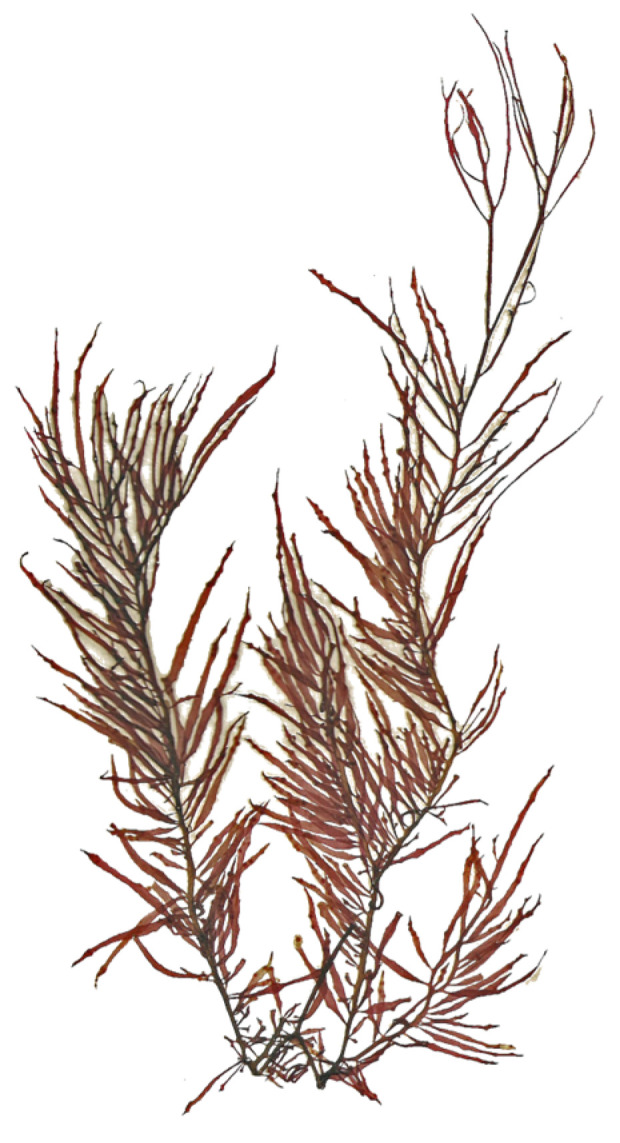
*Solieria chordalis*.

Photosynthetically active radiation (PAR) was found to be the most influential environmental factor on the carrageenan content of Gigartinales seaweeds collected in the field [6]. PAR is one of the main factors determining photosynthesis in macroalgae, and this, in turn, determines the amount of carbon available for carrageenan biosynthesis [6]. Thus, it is not surprising that several studies have found a positive relation between PAR and carrageenan content in various species of seaweeds [6,71,72]. However, in several other studies, high PAR values coincided with lower carrageenan yield [6,31,73,74], where there is photoinhibition. These seemingly opposing findings can be explained by the fact that not only light, but also nutrient availability determines what type of carbohydrate production is favoured [6,16]. In an experiment on *Solieria chordalis* (Figure 7), Fournet et al. [75] found that floridean starch, produced during high-light and low-nutrient conditions, was likely converted into carrageenan during high-light, nutrient-enriched conditions (150 µmol L^−1^ nitrate, 7 µmol L^−1^ phosphate) (cultivation method: filtered seawater with 0 to 49 µmol NO_3_^−^ and 0 ± 1 to 0 ± 8 µmol PO_4_ per liter). With weekly additions of nitrate (NaNO_2_^−^) and phosphate (Na_2_HPO_4_.2H_2_O), seawater was changed once a week. Cool-white, fluorescent lights were used to give an irradiation of 50 µmol photons m^−2^ s^−1^ (400–700 nm) and a photoperiod of 16:8 (light/dark). In addition to interactions with nutrient enrichment, another interaction was observed between light and life cycle phase in an experiment exposing *Mazzaella laminarioides* to various light treatments by Navarro et al. (cultivation method: Provasoli-enriched saltwater [20 mL L^−1^; 31 PSU] was kept in a temperature-controlled chamber at 9  ±  1 °C and artificially irradiated with 55 μmol photons m^2^ s^−1^ PAR, supplied by Philips TLT 20W/54 daylight fluorescent tubes, on a 12–12 h light–dark cycle) [76]. Tetrasporophytes decreased in carrageenan yield under high-light conditions, whereas gametophytes increased carrageenan content in the treatments with UV light, suggesting a protective function of carrageenan in the gametophytes of this seaweed species.

##### Effect of Light

**Figure 8:**
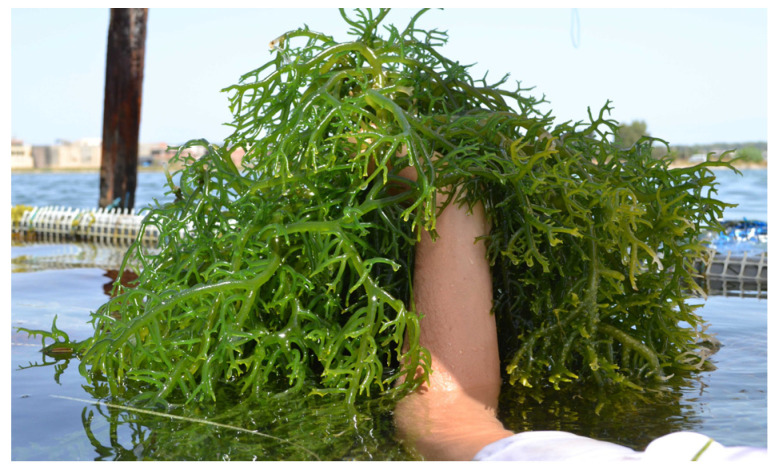
*Kappaphycus alvarezii*.

The influence of light on carrageenan quality seems unclear. Wakibia et al. [72] found gel strength from *Kappaphycus alvarezii* (Figure 8) to coincide with high photon fluency rates. In contrast, Kravchenko et al. [31] found a positive relation between PAR and the number of sulphate groups in carrageenan in *Gymnogongrus flabelliformis* (formerly *Ahnfeltiopsis flabelliformis*) (no cultivation, wild sampling and analysis), with a high amount of sulphate commonly associated with lower gel strength [72]. However, the link between sulphate and gel strength is still controversial, and gel strength is likely increased by more factors like molecule length and 3,6-anhydrogalactose content [72].

From the 1970s onwards, the high impact of nitrogen availability on phycocolloid content in seaweeds has gained a lot of attention [77]. The subsequent concept of the ‘Neish effect’ describes that the carrageenan content of seaweeds in nitrogen-enriched seawater tends to be lower than in unenriched waters [77]. Nutrient addition to starved seaweeds is found to reduce carrageenan content in many studies, and likewise, carrageenan content is often found to be higher with low or no enrichment [77,78]. This is generally thought to be caused by the predominance of protein synthesis during the active growth of seaweeds, whereas the focus shifts to polysaccharide synthesis during N-limitation [79].

Additionally, since a low dry weight sometimes coincides with periods of active growth, the above can explain why carrageenan content sometimes follows the biomass dynamics in seaweeds [31,74]. However, the opposite of the Neish effect is also frequently observed, with carrageenan content being higher in nutrient-rich waters [6,73,75,80]. Chopin et al. [77] note that a slight degree of nitrogen enrichment yields the highest carrageenan content in *Agardhiella subulata*. In addition, Wakibia et al. [72] stress that often inorganic carbon supply is also limiting in seaweeds, making both high growth rate and high carrageenan content possible with an abundance of both nitrogen and carbon.

Besides, analogous to the Neish effect, a ‘P effect’ has also been found for phosphorus [77,81], although phosphate had a slightly lower variable importance in the meta-analysis by Veliz et al. [6]. With temperature influencing virtually every aspect of seaweed functioning [16], it is expected that temperature also influences carrageenan content in seaweeds. In multiple studies, the high carrageenan content of Gigartinales coincides with the warm season, which has been linked to higher rates of photosynthesis and growth [6,71,82]. However, fast growth is not always clearly linked to high carrageenan yields, as shown by the build-up of carrageenan content in various *Eucheuma* species and *Chondus crispus* from summer to winter, coinciding with a stagnated growth [79,83]. In various other species and climate zones (from warm to cold), high carrageenan yields, and sometimes carrageenan quality, have been linked to colder temperatures and seasons [6,15,31,74,78,84]. Maybe, in addition to phylogenetic differences, these opposed trends could be unified by potential interactions with nitrogen availability. In addition, in a laboratory experiment subjecting the tropical species *Kappaphycus alvarezii* to various temperature treatments, carrageenan yield, quality, and viscosity were all negatively impacted by temperatures higher than ambient [15]. Since these effects were accompanied by a reduction in several parameters indicating seaweed health, this experiment might first and foremost demonstrate an upper limit in temperature tolerance, which predicts risks for carrageenan production due to global warming [15]. Salinity is among the less important predictor variables in the meta-analysis of Véliz et al. [6]. In line with this, the literature about salinity is ambiguous. The negative charge of sulphated polysaccharides is hypothesized to play a role in maintaining the ionic equilibrium in seaweeds [4].

##### Effect of Other Abiotic Factors

**Figure 9:**
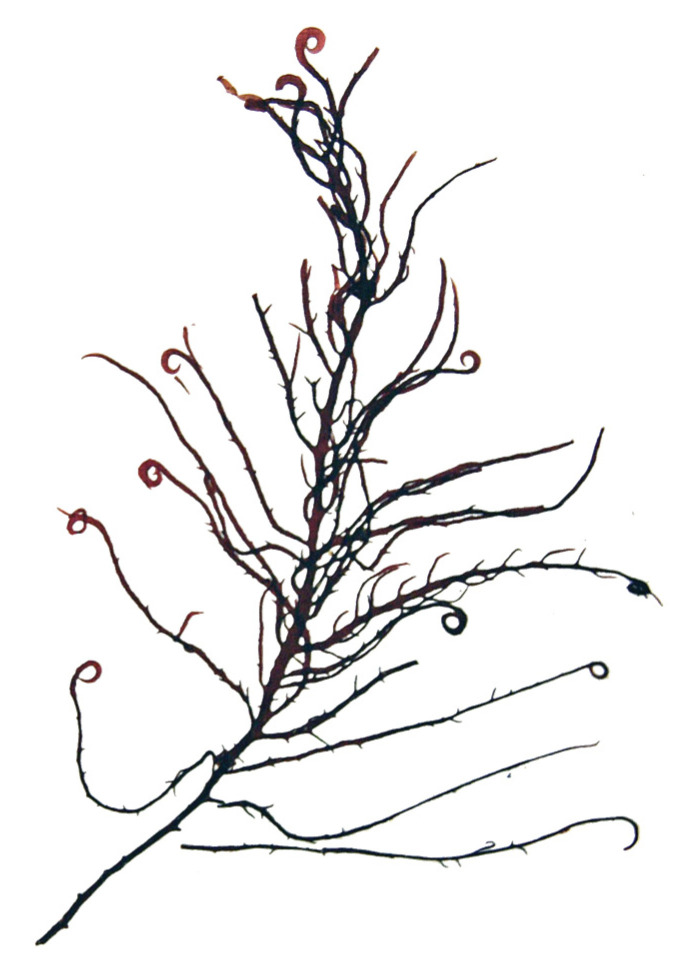
*Hypnea musciformis*.

Following this, *Hypnea musciformis* (Figure 9) carrageenan yield and viscosity increases in a field study are thought to be driven by a higher temperature and salinity [85]. Although salinity increased carrageenan viscosity in *Kappaphycus alvarezii*, it decreased yield [86]. Even more contrasting is a field study on *Hypnea flagelliformis*, which was seen to yield higher carrageenan quantities at lower salinities [84]. Depth was another variable with some predictive power for carrageenan content in the meta-analysis of Véliz et al. (2017), which might be viewed as a product of the effects of desiccation, temperature, light, and other abiotic factors. However, both for *Kappaphycus striatus* (“var. sacol”) and for *Kappaphycus alvarezii*, no effect of depth could be established on carrageenan yield or quality [87,88]. A more recent field experiment on *Kappaphycus alvarezii* growing at depths of 0.2, 1, 2, 3, 4, and 5 m confirms this, even though growth rates varied with depth (offshore farming, floating raft cultivation system) [68]. On the other hand, carrageenan viscosity in *Kappaphycus alvarezii* was lower at a depth of 5 m compared to 2 m depth in a field cultivation experiment, and 64% of the variation in viscosity could be explained by the values of other multiple abiotic factors differing between depths [69].

In the meta-analysis of Véliz et al. [6], exposure to wave-action and water movement is often considered to influence carrageenan content. Carrageenan content and viscosity are thought to increase flexibility in seaweeds and function as structural components as protection against wave-action and water movement [71,72,85,89]. This has led several authors to hypothesize wave-action as a driver for the dynamics of carrageenan quantity and quality they observed [71,72,85,89]. However, water motion also influences the nutrient and carbon supply for seaweeds [16,72], which in turn, can influence polysaccharide content. Additionally, desiccation is also thought to increase carrageenan production [71,85]. By retaining water, carrageenan can help seaweeds survive periods of desiccation from emersion [71,85].

#### 4.3.2. Agar

The other sulfated galactan commonly found in the cell wall of various red algae is agar. It is thought to protect algae against desiccation, temperature, extreme salinity, pH, and pathogens, in addition to maintaining their ionic equilibrium [4,5]. A lot of the research on agar in seaweeds has focused on optimizing extraction methods, as reviewed by Abdul Khalil et al. [11]. Even so, environmental influences on agar content have been studied extensively, too. Trends in this have revealed that agar content is mainly higher in summer, under hypo- and hyper-salinity and light deprivation, while it is lower with nitrogen enrichment and epiphytic growth [5]. An earlier review of the factors influencing agar yield and quality was written by Lee et al. [8]. However, several new studies have been published, which will be discussed here, alongside the main points of the study by Lee et al. [8].

##### Effect of Seasonal Changes of Environmental Conditions

Seasonal changes of environmental conditions influence agar content in seaweeds [8]. Most papers on the subject observe a higher agar content in summer than in winter at temperate latitudes and a higher content in the rainy season than in the dry season at tropical latitudes [8]. The occasionally different observations on this subject are hypothesized to be due to methodological differences, regional effects like habitats and environmental variables, and genetic differences [8,90].

**Figure 10:**
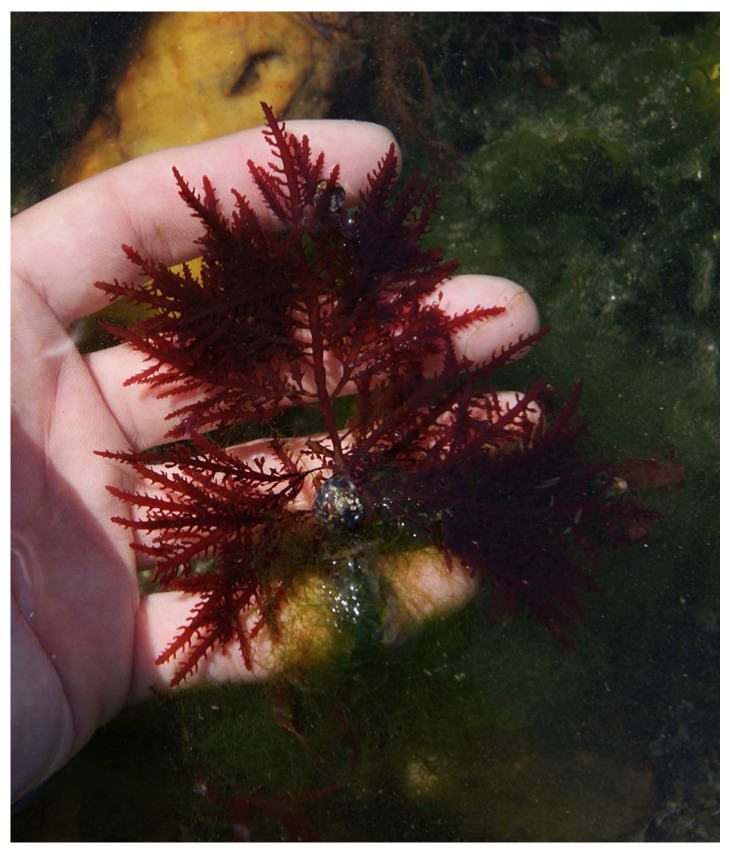
*Gelidium spinosum*.

Several recent studies (wild sampling) are inconsistent with the general seasonal trend as presented by Lee et al. [8]. *Gracilariopsis persica* in Iran [91] and *Gracilaria salicornia* in Kenya [92] yielded constant agar quantities between months. *Gelidium spinosum* (formerly *Gelidium latifolium*) (Figure 10) did not show statistical differences in agar content between seasons either, but varied in several gel quality characteristics, with, for example, gel strength being the lowest in winter and increasing throughout the year [90]. In contrast, the agar quality of *Gracilaria vermiculophylla* (formerly *Agarophyton vermiculophyllum*) was the highest in summer (September) and the lowest in December [93]. However, in various *Hypnea* species in Pakistan, agar yields were the highest in winter, with quality also varying seasonally [94]. *Gracilaria bursa-pastoris* in Tunisia had the highest yield in spring [95]. Additionally, the adelpho-parasite is thought to influence the seasonal trends of agar from *Gracilaria salicornia* in Thailand [96].

##### Effect of Salinity

Salinities lower than 10 ppt and higher than 40 ppt are thought to increase agar yield and slightly lower gel strength [8]. Possibly, the initial amount of floridean starch determines the influence of salinity on agar content, explaining differences between some findings [8]. In a systematic and extensive experiment on the optimal ranges of abiotic factors for *Gracilaria gracilis* cultivation, salinity interacted with ammonium and light in its effect on agar yield (cultivation method: white-light, fluorescent lamps [Philips Actinic BL, TL 15W, Poland] at 7 μmol photons m^−2^ s^−1^ under a 12:12 light/dark photoperiod with different salinities [24, 32, and 40 PSU] were obtained by mixing natural seawater [salinity  =  42 PSU, 180 mg L^−1^ dissolved inorganic carbon, or DIC], pH  =  7.9, NH_4_^+^  = 1 μmol L^−1^, NO_3_^−^  =  16 μmol L^−1^, PO_4_^3−^  =  10 μmol L^−1^ with tap water; germanium dioxide [1 mL L^−1^] was injected to control diatom development) [97]. Low salinity in combination with low light yielded more agar, which was hypothesized to be due to the degradation of floridean starch under these circumstances [97]. However, in its interaction with ammonium, high salinity and low ammonium levels yielded more agar [97]. Since the latter was a more drastic effect, optimal agar yield was found to coincide with a high salinity [97]. High salinity has also been related to increased agar gel strength and gelling temperature and decreased melting temperature in *Gracilaria debilis* [98]. The seaweed species and the impact of other abiotic and biotic factors can differentiate the results of the studies; thus, this is why it is important to conduct seaweed cultivation assays for polysaccharide extraction, to better manage and control the polysaccharide.

##### Effect of Light Deprivation

Light deprivation results in higher agar contents in *Gracilaria* and *Gracilariopsis* species, whereas high-light conditions increase starch content [8], a compound that can be converted to agar. Gel strength is enhanced, and L-galactose-6-sulphate content is lowered under low-light conditions, an effect that is enhanced at high temperatures [99]. Additionally, altered salinities, in combination with light deprivation, are thought to increase the degradation of Floridean starch and, thus, yield higher agar quantities [8]. Affirming this, in the experiment into the optimal growth conditions of *Gracilaria gracilis* by Fethi and Ghedifa [97], agar production was the highest with high salinity and low light.

However, in another factorial experiment on *Gracilaria corticata*, no significant effect of irradiance on agar yield was found [100]. Moreover, in the field, alongside a geographical gradient in Chilean *Gelidium lingulatum*, there was no correlation between daily photosynthetically active radiation (PAR) and agar content (wild sampling) [101]. This might be due to potential interactions with light and other factors such as salinity, as found by Fethi and Ghedifa [97].

The relation between light conditions and agar yield can also be investigated based on depth, which causes lower light conditions. In the Chinese *Gracilariopsis lemaneiformis* (formerly *Gracilaria lemaneiformis*), greater depth resulted in higher agar content [102], which is in line with the effect of light-deprivation experiments [8]. However, *Gracilaria gracilis* in Tunisia was found to yield more agar with a higher melting temperature at 0.5 m depth, although gel strength and gelling temperature were higher at 2.5 m [103]. Besides depth, a high sedimentation level also creates lower light conditions, which reduces photosynthesis and, thus, reduces the agar yield when compared to no sediment [8,104].

##### Effect of Temperature

Temperature is possibly one of the main drivers of the seasonal changes of agar content, as the agar yield increases throughout the summer (correlated with water temperature) [8]. Gel strength is sometimes found to follow this trend, but also might be lowered by temperatures higher or lower than ambient [8]; moreover, agar yields decrease with high temperatures during summer or rapid growth [8], suggesting that some upper-temperature optimum exists. This might explain the fact that the agar yield was higher in *Gracilariopsis persica* at temperatures of 17.45–22.5 °C, whereas it was lower at higher temperatures [91]. However, alongside a large geographical gradient of daily sea surface temperature (SST) in Chile, no correlation with agar yield was found in *Gelidium lingulatum* [101]. Moreover, in a factorial experiment on *Gracilaria corticata*, temperature did not significantly affect agar yield [100]. Nevertheless, some gel quality parameters have been found to be influenced by temperature: *Gracilaria debilis* gel strength is positively correlated with SST [98], and in *Gelidium spinosum*, viscosity and gelling temperature are thought to be influenced by water temperature [90].

##### Effect of Nitrogen Availability

Generally, nitrogen availability (of either the environment or seaweed tissue) has an inverse relationship with agar content in *Gracilaria* and *Gelidium* species [8]. In *Gracilaria tenuistipitata* var. *liui*, this inverse relationship with dissolved organic nitrogen (DIN) is also found, with gel strength positively correlating to DIN; remarkably, in *Gracilaria fisheri*, the exact opposite relation was found [105]. The negative correlation between agar and nitrogen availability is probably due to seaweeds favouring protein synthesis during high nitrogen conditions [8]. Generally, there seems to be an inverse relation between agar yield and nitrogen, which has been observed in several studies [90,98,106]. In accordance with this, gel strength and melting temperature were generally found to be higher with high nitrogen availability [8]. More recently, affirming the above, the gel strength of *Gracilaria debilis* agar was also found to be positively influenced by nitrogen levels, whereas nitrite had a negative effect on agar yield [98]. In the agarophyte *Gracilariopsis lemaneiformis*, nitrogen deficiency did not cause a difference in the regulation of a molecular marker for agar content (UDP-glucose pyro-phosphorylase), but after four days of nitrogen recovery, this marker was upregulated drastically, coinciding with an increased soluble polysaccharide content [107]. Seasonal agar fluctuations in a field study on *Gracilaria salicornia* were also explained by DIN fluctuations [108]. In the optimum-ranges experiment by Fethi and Ghedifa [97], ammonium interacted with salinity, with high salinity and ammonium starvation yielding higher agar contents. However, this trend was not the same for nitrate, which highlights that seaweeds tend to use different nitrogen sources differently [16,97]. Moreover, it highlights the interactions between nitrogen availability and other abiotic factors. Additionally, ammonium phosphate was found to increase agar yield in *Gracilaria corticata* [100]. The influence of phosphate concentrations on agar content has not been studied extensively. Lee et al. [8] note a decreased gel strength at lower phosphate concentrations. Orthophosphate (PO_4_^3−^) concentration was strongly correlated to agar yield in one locality of a field study of Gracilaria bursa-pastoris in Tunisia, and gel strength in two localities [95].

##### Effect of Sulphate and pH Concentration

The effect of low sulfate concentrations depends on the length of treatment, with three weeks of deprivation reducing agar content, but five days slightly increasing agar yield and gel strength [8]. Additionally, sulfate content in the agar of *G. salicornia* was found to be reduced after sulfate deprivation, but not in *Gracilaria changii* [109]. Besides that, it appears that the literature on this subject remains scarce. At the time of the review by Lee et al. [8], the influence of pH on agar production in seaweeds was still unknown. Fortunately, now its influence has been studied in an experiment with *Gracilaria changii* [110], establishing that both lower (6.61) and higher (9.30) pH values than normal (8.04) increase agar yield. This might be in order to protect the seaweeds from the stress deviations in pH cause [110]. Additionally, gel strength was weaker after a treatment with a pH of 9.30 than the other treatments, but gelling temperature was lower after a treatment with pH 6.61 [110].

##### Effect of Wave Exposure/Water Movement

**Figure 11:**
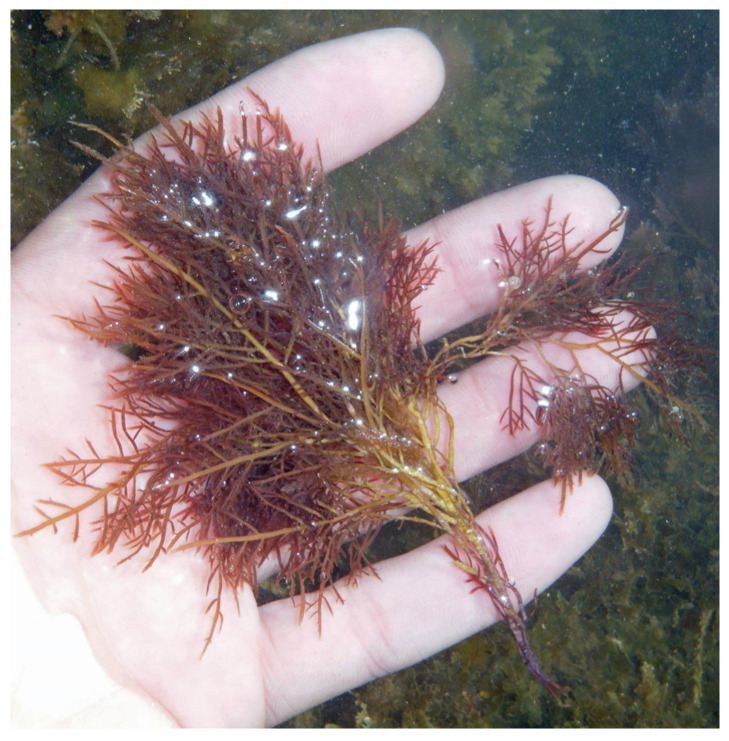
*Pterocladiella capillacea*.

Wave exposure might also increase agar content, as hypothesized for *Pterocladiella capillacea* (formerly *Pterocladia capillacea*) (Figure 11) in a field study [111]. However, this trend was not confirmed by a study investigating the tube-net cultivation method for *Gracilaria dura* [106]. Different diameters of the tube-nets were found to induce different levels of water movement, which resulted in different growth rates, but had no influence on agar quantity or quality [106].

##### Effect of Life Stage and Epiphytes/Epibionts

Lastly, agar yield is also influenced by biotic factors. The life stage is thought to have an influence on agar content and quality, but this is mostly specific for different species [8]. Additionally, epiphytes and epibionts may lower agar content in *Pterocladiella capillacea* [112].

The results presented by Inácio et al. [113] and Mendes et al. [114] support the importance of the cultivation of *Gracilaria gracilis* in aquaculture systems at estuarine adjacent terrains, maintaining the nutritional quality of the algae when compared to wild specimens obtained at coastal marine zones, thus promoting an eco-sustainable way to cultivate seaweed for nutritious food purposes and promote global food security.

### 4.4. Additional Considerations

Elucidating the effects of separate environmental factors on polysaccharide quantity and quality is not straightforward. Besides the fact that correlations in field studies do not necessarily indicate causal relations, the matters are further complicated by interactions between various factors. For example, nitrogen uptake, which is thought to have an influence on the content of some polysaccharides, not only is determined by the concentrations of different nitrogen sources, but also interacts with water motion, light conditions, temperature, carbon dioxide sources, probably salinity, and desiccation [16]. Moreover, besides differences between species, age classes within the same species also display different nutrient uptake rates [16]. In accordance with this, the dynamics of polysaccharide yield in the seaweed studied by Tasende et al. [71] was best explained by interactions between multiple environmental factors. All in all, to truly understand the drivers of polysaccharide production, extensive factorial experiments are needed for different seaweeds and sometimes life stages; for an example, see the study by Fethi and Ghedifa [97].

External factors have great importance for polysaccharide production; however, studies have found that the same factor can have distinct results and react differently with other factors. Thus, it is important to note that to obtain the high yields of polysaccharide with the same quality, it needs to be practical work, with the targeted seaweed in the specific location, so that there will not be a theoretical common factor relation adjusted for the seaweed’s polysaccharide production. This is similar to seaweed phenolic compound production, wherein the high salinity in Australia and Portugal has an inverse effect on the phenolic compound production [61]. Moreover, as demonstrated above, the results and inputs from the studies can be due to different cultivation methods and whether they involved lab scale, inshore, or offshore cultivation. Also, the nutrients in the studies are mostly different, and in wild and field sampling, there is no control over some important variables. Thus, these variations can give inverse inputs to studies involving assays on the same effects.

However, wild and field sampling can have a bigger role in understanding the real behaviour of the seaweeds in that location, which is very important for seaweed farming.

## 5. Extraction Technologies and Safety Measures for Phycocolloid Production

Phycocolloids including agar, carrageenan, and alginate are derived from seaweed using a variety of methods. Ensuring safety during extraction is critical for protecting workers and the environment while also producing high-quality, safe goods. By utilizing these extraction methods, along with security measures, phycocolloid extraction may be performed effectively and safely, assuring high-quality goods while also protecting workers and the environment.

### 5.1. Industrial Extraction Methods

The industry’s traditional method of phycocolloid production involves a multi-stage processing procedure that includes a few key phases like cleaning/washing, pre-treatment, solid/liquid separation (extraction), precipitation, filtration, drying, and milling [11,42]. The existing methods require high chemical, water, and energy consumption, as well as control of waste generated throughout the whole process, which is less environmentally friendly and cost ineffective [11]. Taking all this into consideration, green extraction methods such as ultrasound-assisted extraction (UAE), microwave-assisted extraction (MAE), enzyme-assisted extraction (EAE), supercritical fluid extraction (SFE), and pressurized solvent extraction (PSE), reactive extrusion and photo-bleaching methods are believed to help reduce the chemical usage and improve the extraction yield and quality of seaweed-derived polymers.

#### 5.1.1. Agar

The agar extraction process in the industry requires a high consumption of solvent and produces a large quantity of waste disposal, being a time-consuming process that applies conventional heating that uses hot water during several hours. Acid pre-hydrolysis has been commonly employed for the chemical liquefaction of agarose [115]. The generation of effluents with environmental impact if not properly treated is another disadvantage of alkali treatment. A more eco-friendly alternative to improve the gel strength of agar could be enzymatic treatment; however, the cost could not make the process commercially competitive compared to alkaline treatment [116]. Processes based on combined heat and ultrasound treatments would enable the reduction of the amount of time and energy needed [11]. Microwave-assisted extraction (MAE) allows the reduction of the required time to a very short period for agar extraction in conventional processes, consuming less energy and solvent volume and reducing waste disposal requirements [42].

#### 5.1.2. Carrageenan

The original method of producing the commercial carrageenans is based on washing to remove impurities, such as sand, epiphytes, and salt, and extracting the carrageenan in a hot aqueous solution, neutral or alkaline, then filtrating, recovering it from the solution by alcohol precipitation, recovering the precipitate, drying, and milling. Depending on the extraction method, carrageenan can be classified into two distinct grades known as semi-refined carrageenan (SRC) and refined carrageenan (RC) [117]. In the original method, in the late 1970s and early 1980s, the carrageenan was extracted from the seaweed into an aqueous solution. Carrageenan is frequently extracted from the seaweed by alkali treatment, and the result is referred to as SRC at this point in the process [118]. Additional treatments, such as filtering and purifying procedures, are necessary to eliminate residual components such as cellulosic materials, yielding RC [117]. Other options can reduce the time, energy demand, and consumption of water, chemicals, and solvents. Among the novel extraction techniques to enhance the extraction efficiency are pressurized solvent extraction and microwave-, ultrasonic-, and enzyme-assisted extractions [42]. Microwave-assisted extraction offers a reduction in time and energy consumption, thus enhancing the process efficiency [42]. The ultrasound-assisted processes, both alkaline and aqueous, shorten extraction times compared to the conventional method, avoiding the degradation of labile compounds and showing a slight variation in sulfate and viscosity.

### 5.2. Industrial Safety Measures

Since polysaccharides present varied compositions, which can be attributed to the extraction process or to abiotic and biotic factors, there is a need to use chemical and biochemical methods to guarantee their quality.

Chromatography is a physical method of separation in which the components of a mixture are separated by their distribution between two phases; one of these phases, in the form of a porous bed, bulk liquid, layer, or film, is generally immobile (stationary phase), while the other is a fluid (mobile phase) that percolates through or over the stationary phase [119]. Chromatography follows a wide range of techniques that can be applied in a sequential way to isolate with an excellent rate and high efficiency in the characterization of seaweed quality; however, these techniques are often costly [120]. Nevertheless, there are other methods of chromatography, such as high-performance anion exchange chromatography (HPAEC), that can be used to quantify and characterise seaweed polysaccharide fraction [121], which is the most difficult to analyse by liquid extracts due to its viscous properties [122]. This method uses a strong anion exchange to separate the fractions by pH and the acidic nature of the seaweed polysaccharides (seaweed carbohydrates) [123].

Spectroscopy techniques use infrared light frequencies to analyse light absorbance by the sample and vibration by chemical bonds. Fourier transform infrared spectroscopy (FTIR) is a useful technique with low cost compared to other techniques (e.g., NMR, X- ray, chromatography) analysing the chemical bonds of dried samples, in opposition to the above techniques, which require a liquid extract solution form [29]. This technique can be used to see the polysaccharide, pigment and phenolic fractions, compound oxidation, and microplastic in the seaweed before commercialization [22,124,125,126]. The FTIR technique cost is established according to the technician and the equipment, being cheaper and easier to operate when compared to chromatography; however, the biochemical quantification and quality analysis are not so good. However, accoupled techniques can provide an enhancement of seaweed quality [127]. Pereira and respective team [20,29] employed FTIR–ATR (attenuated total reflectance) spectroscopy, which allowed the determination of the composition of different phycocolloids by analysing dried ground seaweed, without having to prepare tablets of KBr [29]. Therefore, FTIR–ATR can be, and is, applied to differentiate the agar and carrageenan quality by the seaweed polysaccharide extraction industry (the analysis is between the extraction sample and sample reference). Also, FTIR spectroscopy is the most efficient and ecologically benign technology for analyzing biomass. The three primary uses are identification, quality control, and structural elucidation. The IR technique is utilized in industrial sectors to quickly detect significant properties in order to approve chemicals such as phycocolloids and active ingredients for pharmaceutical medications. In this specific case, the FTIR spectra are applied to differentiate between agar-producing and carrageenan-producing seaweeds [128,129,130].

All types of chemical analysis have advantages and disadvantages when it comes to certifying seaweed quality. The currently used techniques are not the most appropriate ones, with some using methods applied to plants (ignoring the mandatory heavy metal checks). There is still a need for the development and improvement of these techniques (which is a challenging due to the complexity of seaweed composition) without imposing high costs on the seaweed companies. Thus, more work is needed at the legislative level for seaweed food quality control checks due to the chemical variability in seaweeds and their compound complexity [17].

### 5.3. Technical Characteristics of Agar and Carrageenan

Agar and carrageenans are frequently utilized in food, medicines, and biotechnology because of their unusual gelling, thickening, and stabilizing qualities. Their technical features need to be analysed and approved for consideration as polysaccharides by CAS and other national and international agencies, to be applied in the industry with safety and security.

#### 5.3.1. Agar

Agar powder either is odourless or has a faint odour. Unground agar is typically found in packages of thin, membranous, agglutinated strands, as well as sliced, flaked, or granular forms. It can vary from light yellowish-orange to yellowish-grey to pale yellow, or it can be colourless. When wet, it is tougher and forms a jelly type; when dry, the agar is very fragile. Agar powder presents a white/yellowish-white or light golden colour. Agar powder looks more transparent when seen under a microscope in water. Powdered agar in chloral hydrate solution appears even more transparent than in water, more or less granular, striated, and angular, and occasionally contains diatom frustules [131].

Agar is insoluble in cold water and soluble in hot water. Its purity level is considered by ash content (food-grade ash below 6.5%); insoluble matter; detection of starch, lipids, and proteins; water absorption capacity; and viscosity [131].

Thus, there are several techniques similar to other polymers’ biochemical and physical–chemical characterization and certification methods, for example, glucans and xanthan gums [132].

#### 5.3.2. Carrageenan

Carrageenan powder is normally a yellowish to colourless, coarse to fine powder that is practically odorless. To be considered carrageenans (by food agencies worldwide), carrageenans need to have galactose, anhydro-galactose, and sulphates. If one of the molecules is not identified in the sample, it is not considered carrageenan. It is only soluble in hot water and not soluble in alcohol solution above 1.5% [133].

For the food purity rate of carrageenan, it is considered that the most important factors are the viscosity level (5 mPa·s [1.5% solution at 75 °C]); sulphation content (between 15% and 40% carrageenan DW); ash yield (between 15% and 40% carrageenan DW); low molecular-weight carrageenan content (molecular weight fraction below 50 kDa, where over 5% is considered a poligeenan [a non-approved polymer for the food industry]); detection of starch, lipid, and protein levels below 5% (between them all); and high water absorption capacity [133]. Thus, there are several methods similar to other polymers’ biochemical and physical–chemical characterization methods [132].

This has led to RD units and industry which wants to innovate and exploit new red seaweed polysaccharide extractions with more eco-friendly processes and purification techniques. Thus, the results of this continuous research need to demonstrate that the polymers have high purity according to CAS and international conventions [62,134]. That is essential to certify the quality of the compound to be further exploited.

### 5.4. RD of Polysacharide Extraction Methods

Although the phycocolloid extraction industry has implemented certified methods, these methods are labour-intensive, have medium to low extraction rates, use substantial amounts of chemicals, water, and energy, and generate waste during the process, making them less environmentally and economically efficient. Given all of this, numerous green extraction techniques are emerging to decrease chemical use while enhancing the extraction yield and quality of seaweed-derived polymers. However, they need to pass the tests and the requirements for industrial polysaccharide commercial samples, and only after this can they be applied to the industry. However, most of the studies are not conceived for industrial scale; even with the results, this is another important factor of the economic feasibility of the extraction process [17,135,136].

These new techniques include microwave-assisted extraction (MAE), ultrasound-assisted extraction (UAE), enzymatic-assisted extraction (EAE), and green solvent extraction, which includes subcritical water extraction (SWE), ionic liquid extraction, supercritical fluid extraction (SFE), and other methods like reactive extrusion and photo-bleaching. There is less information accessible due to the several distinct methods that are currently in use. Currently, MAE and UAE procedures are low-cost and have been effectively used in large-scale commercial chemical extractions [11,135,137,138].

Also, polysaccharides have a variable composition, which can be related to the extraction process or to abiotic and biotic factors, thus many procedures must be used to describe them and ensure their quality. Finding new, effective, and environmentally acceptable extraction technologies for commercial scale is critical. Hydrocolloids, like carrageenan and agar, are often processed inefficiently using significant amounts of chemicals; nevertheless, there are innovative and equally acceptable alternatives to meet industrial productivity demands [135].

As a result, while researching novel species and cultivation and extraction procedures for obtaining carrageenan and agar for industry approval, it is critical to investigate the identification and purity of the material to ensure its safety and approval by regulatory requirements. Mendes [135] demonstrated that different species strains (*K. alvarezii*) can influence the right method to extract carrageenan, which can be approved for food application. Thus, the effect of different extraction procedures on the kind of extracted carrageenan was very clean.

## 6. Industrial Innovation: Emerging Applications of Seaweed Polysaccharides

The phycocolloid industry is now increasing at a faster pace than the world GDP, at 6% per year. While phycocolloids have historically been employed as food additives in the food and beverage sector, which accounts for more than 70% of their worldwide market value, their usage in personal care, cosmetics, and the medical and pharmaceutical industries is likely to grow the quickest [139].

With emerging knowledge and technological advancement, the applications of algal polysaccharides now start to stretch beyond the common applications discussed above. Here, applications that are still being studied or are new to the industries will be discussed. Firstly, the antiviral activity of several seaweed polysaccharides has inspired an exploration of their potential in battling the global COVID-19 disease [49]. Algal polysaccharides are argued to help to prevent the attachment, adsorption, and replication of the virus [49]. Moreover, their safety, biodegradability, and biocompatibility, alongside their cheap production costs, potentially give them advantages over plant-based compounds [49]. Also, another advantage of the red seaweed polysaccharides over the plant-based compounds is their inherent bioactivity potential. As previously stated, certain polysaccharides, such as agar and carrageenan, exhibit a variety of biological activities; nevertheless, their biological activity differs based on their molecular weight, sulphation level, and the number of sulfate esters groups present in the polymer theta [30,140]. Furthermore, various seaweed species generate chemically diverse polysaccharides, which might potentially be influenced by the extraction process used. Carrageenophytes often possess a larger concentration of sulfate groups than agarophytes [141]. Nonetheless, carrageenan’s molecular structure is quite diverse, with the most economically important carrageenan iota, lambda, and kappa [37]. So, what distinguishes these distinct forms of carrageenan is primarily their sulfate ester concentration and their location in the molecule [142].

### 6.1. Pharmaceutical Applications

Moreover, the nanoparticles of seaweed polysaccharides have attracted attention for several other therapeutical applications. Drug delivery, insulin delivery, and bioactivities against fungal infection, tuberculosis, and cancer are some of the applications that are being explored [48].

Carrageenan has sparked widespread attention, and its use in pharmaceutical formulations is on the rise. It has been included in respected pharmacopoeias such as the United States Pharmacopeia 35-National Formulary 30 S1 (USP35-NF30 S1), the British Pharmacopoeia 2012 (BP2012), and the European Pharmacopoeia 7.0 (EP7.0), suggesting its potential as a pharmaceutical excipient and a bright future. Carrageenan has been shown to have antiviral and antibacterial characteristics, as well as anticoagulant, anti-diabetic, and antioxidant action. There is a rising interest in combining carrageenan with natural polymers such as chitosan, starch, cellulose, chitin, and alginate to develop biodegradable materials with desirable properties for use in biomedical applications. These combinations have demonstrated tremendous potential in a variety of biological applications, including drug delivery and tissue engineering [143,144,145]. Carrageenans’ outstanding bio-functionality and rheological properties, including cost-effectiveness, biological compatibility, biodegradability, and flexibility, make them ideal functional compounds for a wide range of biomedical applications, from developing nanostructure-based intelligent drug delivery systems to 3D bioprinting in tissue engineering and wound healing [139].

Agar is increasingly preferred over synthetic polymers and is being investigated as an alternative raw material for therapeutic purposes. It is very desirable in the pharmaceutical industry because of its remarkable intrinsic properties, notably the strong gel it produces. Agar has been exploited in the creation of injectable and phase-changeable composite hydrogels for cancer treatment using chemotherapy and photothermal therapy. These composite hydrogels can efficiently load and release chemotherapeutics and antibiotics. Additionally, an agar-based nanocomposite film has been shown to effectively suppress the development of *Listeria monocytogenes*. The usage of agar and polysaccharide mixes is also becoming common. In pharmaceuticals, agar is used primarily to gel, stabilize, and thicken. Furthermore, it is often used for purgation and as a surgical assistance. Researchers have worked hard to develop agar-based products such as composite hydrogels, nanocomposite films, and other materials with specialized applications in pharmacology [143,146].

### 6.2. Food Packaging

Additionally, food packaging is important for the preservation, protection, and convenience of food products, but is often not done sustainably. However, polysaccharide membranes can work as a barrier against oxygen and carbon dioxide, making them a promising candidate for food packaging [147]. Moreover, they are edible and biodegradable, so the environment is not impacted whether people eat the packaging or not. However, they do not work as a barrier for water or water vapor due to their hydrophilic properties, which can become a problem for durability and change the organoleptic characteristics of the product [147]. A method to mitigate the risks of spoiled food is intelligent food packaging. This concept focuses on the observance and control of changes in the food or in the food packaging. It is done using biosensors of hydrogel made from polysaccharides to measure those parameters [148].

For example, it was demonstrated that the spoilage of fish could be slowed by using a partly agarose-based hydrogel [149]. Another utility with polysaccharides is making biostimulants for plant growth. In a study in which polysaccharide extract was given to growing plants, all the evaluated parameters (total plant size, leaf growth, and weight) were further developed in the plants with the treatment [150,151].

Seaweed polysaccharides’ adaptability allows for a wide range of unique uses across many sectors. As research and development continue, the usage of these sustainable and bioactive molecules is anticipated to grow, resulting in novel solutions in health care, environmental sustainability, and beyond. However, these novel applications are still in their early stages, with R&D for the methods in its initial steps and with significant restrictions and difficulties to overcome in the immediate future. Furthermore, the cost-effectiveness analysis of scaling-up still needs to be applied, to check if it is possible to be applied at the industrial scale.

## 7. Future Road for Red Seaweed Polysaccharide Exploitation

Considering the actual blue and circular economic paradigm of the economy, there is a new goal in the agar and carrageenan extraction industries, with the possibility to scale-up and develop green extractions and recover techniques. This concept of a circular economy is focused on the biorefinery concept, which involves the integration of biomass into the production of various value-added products/compounds (according to the biomass quality), reducing the total waste at the industrial level [152,153]. Also, the biorefinery conceptualization is based on a multi-solution strategy, obtaining low-volume, high-value-added products and vice versa, ending the cycle. The cascade valorization strategy (including the selective, sequential extraction of value-added chemicals), paired with the generation of biofuels or soil amendments, may be considered within the biorefinery idea [153].

Thus, red seaweed has interesting compounds that can be exploited before polysaccharide extraction. After meeting safety requirements, several high-value pigments and bioactive compounds are employed in the biomedical and food industries [153]. Although commercial extraction methods for some pigments (e.g., fucoxanthin and phycoerythrin) have previously been created, industrial extraction methods are still being developed [17]. If biorefinery is used from the start, the seaweed biomass can be explored with greater value, reducing pre-treatments (which are currently used to clean the polysaccharides at the industrial level) and manufacturing costs [154].

The final waste from the biorefinery concept mainly includes ashes and minerals, which can be applied to nourish the soil for agriculture [153], thus creating a near zero-waste industry. However, this is still in its initial steps, although with good results already having been demonstrated at a small scale [154].

However, although there is currently a paucity of knowledge regarding the biochemical variety of seaweed polysaccharides produced by cultivated seaweeds, we need to exploit the seaweeds to obtain a good yield with good quality, as well as the seaweed’s mechanisms of action for certain functions, which can be different in different locations. All investigations on this fail to address the relationship between seaweed polysaccharide with particular abiotic and biotic components and the yield, structure, and molecular length of basic monomers of agar and carrageenan. Due to the industry, the polysaccharide quality is very important.

## 8. Conclusions

Carrageenan content and quality are affected by several environmental factors. The impact of photosynthetically active radiation (PAR) is likely determined by its interaction with nutrient availability, where high light in combination with low nutrients yields high carrageenan content. Additionally, the effect of light sometimes interacts with the seaweed’s life stage, and carrageenan gel strength might be positively affected by light intensity.

Several factors can influence polysaccharide production in seaweeds, as demonstrated in Table 2.

Without a doubt, the exact influences of many factors on polysaccharides in seaweeds remain unclear. Not only are there differences in polysaccharide dynamics between species and life stages; interacting factors further obscure the situation. Another point to consider is that for polysaccharide production, a balance must be found between high seaweed growth and high polysaccharide quality and quantity. All in all, more factorial experiments are needed for different species and sometimes life stages. However, the knowledge gathered above represents a step towards understanding polysaccharide production in seaweeds. It may inform aquaculture and harvesting methods when obtaining seaweed polysaccharides, which have been hindered by inconsistent yields and difficulty in optimization. The range of current and developing applications for seaweed polysaccharides and the abundance of research on the matter show the relevance of these interesting compounds for both science and society.

## Figures and Tables

**Table 1:** Résumé of the red seaweed polysaccharides’ industrial applications.

Industry	Polysaccharide	Main Applications	Specific Uses
Food Industry	Agar	- Food additive (E406, GRAS approved)	- Low-quality agar in food products
		- Popular in jellies	- High-quality agar in limited food items
	Carrageenan	- Food additive (E407, GRAS approved)	- Common use as a jellifying agent
		- Processed meat products stabilization	- Binds milk molecules, retains water
		- Protective coating on fresh-cut packaged food	- Gas barrier, reduces respiration, slows discoloration, and maintains texture in packaged foods
Pharmaceutical	Agar	- Pharmaceutical-grade growth media	- Decreases blood glucose, prevents red blood cell aggregation- Acts as bulking agents in laxatives, suppositories, capsules, tablets, and anticoagulants
		- Drug delivery systems	- Production and encapsulation of monoclonal antibodies, interferons, steroids, and alkaloids
		- Functional foods with health benefits	- Decreases blood glucose, prevents red blood cell aggregation
		- Medical analysis	- Highly purified agar (agarose) used in molecular biology (electrophoresis, immune diffusion, gel chromatography)
	Carrageenan	- Pharmaceutical drugs and agents	- Tetracycline production (immobilizes bacteria for antibiotic production)- Produces D-aspartic acid for semi-synthetic antibiotics- Inhibits viruses like human papillomavirus, dengue, influenza A, and herpes virus
		- Functional foods with health benefits	- Cholesterol-lowering effects, immunomodulatory activity, and antioxidant activities
Cosmetic	Agar	- Structural ingredient	- Used in creams, hand lotions, liquid soap, deodorants, foundation, exfoliant, cleanser, shaving cream, face moisturizer/lotion, acne and anti-aging treatments
	Carrageenan	- Structural ingredient	- Applications include toothpastes, hair wash products, lotions, medications, sun blocks, shaving creams, deodorant sticks, sprays, and foams
Agriculture	Agar	- Structural ingredient	- Acts as moisture-holding hydrogel, reducing irrigation frequency, improving soil aeration, and limiting erosion- Soil conditioner, improves water retention, soil permeability, and plant performance
	Carrageenan	- Functional ingredient	- Regulates plant metabolic processes, including purine and pyrimidine synthesis, nitrogen and sulphur absorption- Activates plant defense systems, provides resistance to abiotic and biotic stressors
		- Regulates physiological and biochemical processes in plants (e.g., cell division, photosynthesis)	

**Table 2:** Factors that can influence the polysaccharide production on seaweeds.

Factor	Effect on Carrageenan	Effect on Agar
Nitrogen (high concentrations)	Inverse relationship with carrageenan content (Neish effect)	Inverse relationship with agar content; higher nitrogen availability may increase gel strength
Phosphorus	Probably inverse relationship with carrageenan content	Phosphate may increase gel strength and possibly yield
Temperature	Detrimental effects above thermal tolerance on quantity and quality	Generally positive relation with agar content up
Salinity	No clear pattern	Positive effect of both high and low salinities, although not unanimous
Depth	No significant effect on content	Effect on agar remains unresolved
Water Motion (Wave Action)	Possible influence on carrageenan content	Inconclusive effects on agar content
Carbon Sources	may enhance carrageenan content under nitrogen enrichment	Not specifically mentioned
pH	Not specifically mentioned	Not specifically mentioned
Sulphate Deprivation	Not specifically mentioned	May reduce agar content

## Data Availability

Not applicable.
